# Influence of age and sex on longitudinal metabolic profiles and body weight trajectories in the UK Biobank

**DOI:** 10.1093/ije/dyae055

**Published:** 2024-04-19

**Authors:** Ville-Petteri Mäkinen, Mika Ala-Korpela

**Affiliations:** Systems Epidemiology, Research Unit of Population Health, Faculty of Medicine, University of Oulu, Oulu, Finland; Biocenter Oulu, University of Oulu, Oulu, Finland; Systems Epidemiology, Research Unit of Population Health, Faculty of Medicine, University of Oulu, Oulu, Finland; Biocenter Oulu, University of Oulu, Oulu, Finland; NMR Metabolomics Laboratory, School of Pharmacy, University of Eastern Finland, Kuopio, Finland

**Keywords:** Ageing, metabolism, metabolomics, longitudinal, menopause, sex difference, body mass index, waist-hip ratio

## Abstract

**Background:**

Accurate characterization of how age influences body weight and metabolism at different stages of life is important for understanding ageing processes. Here, we explore observational longitudinal associations between metabolic health and weight from the fifth to the seventh decade of life, using carefully adjusted statistical designs.

**Methods:**

Body measures and biochemical data from blood and urine (220 measures) across two visits were available from 10 104 UK Biobank participants. Participants were divided into stable (within ±4% per decade), weight loss and weight gain categories. Final subgroups were metabolically matched at baseline (48% women, follow-up 4.3 years, ages 41–70; *n* = 3368 per subgroup) and further stratified by the median age of 59.3 years and sex.

**Results:**

Pulse pressure, haemoglobin A1c and cystatin-C tracked ageing consistently (*P *< 0.0001). In women under 59, age-associated increases in citrate, pyruvate, alkaline phosphatase and calcium were observed along with adverse changes across lipoprotein measures, fatty acid species and liver enzymes (*P *< 0.0001). Principal component analysis revealed a qualitative sex difference in the temporal relationship between body weight and metabolism: weight loss was not associated with systemic metabolic improvement in women, whereas both age strata converged consistently towards beneficial (weight loss) or adverse (weight gain) phenotypes in men.

**Conclusions:**

We report longitudinal ageing trends for 220 metabolic measures in absolute concentrations, many of which have not been described for older individuals before. Our results also revealed a fundamental dynamic sex divergence that we speculate is caused by menopause-driven metabolic deterioration in women.

Key Messages

- Longitudinal changes in 220 metabolic measures were compared between carefully matched subgroups of weight loss, stable weight and weight gain among 10 104 middle-aged and older participants from the UK Biobank.
- We found that maintaining stable weight may not be enough for the female population aged over 40 to avoid the rapid decline in metabolic health (due to menopause), whereas men appear to respond consistently to weight change even when older (weight loss improved metabolic health).
- Our results highlight the need and opportunities to address modifiable cardiometabolic risk factors such as obesity from a sex-specific viewpoint in ageing populations.

## Introduction

Age-associated disease burden increases rapidly during the sixth decade of life.^1^ Biological ageing can be observed as functional decline in physiology brought about by structural and molecular changes in the body,^2–4^ and this phenomenon is trackable at population scale thanks to quantitative molecular profiling.^5–8^ Blood-based nuclear magnetic resonance (NMR) metabolomics has matured as a cost-effective platform for large cohorts such as the UK Biobank.^9^ Although numerous cross-sectional and prospective studies have been published about metabolomics and age-associated morbidity,^10–12^ the temporal trajectories of metabolic measures are rarely characterized using longitudinal data.^13^^,^^14^ Our aim is to fill the gap in knowledge of metabolic ageing for the population segment between the ages 41 and 70, using longitudinal data and carefully adjusted statistical design.

Longitudinal studies yield stronger epidemiological evidence compared with cross-sectional surveys.^15^ Recently, new metabolomics data were released in the UK Biobank, i.e. the Nightingale Health Biobank Collaborative Group [https://doi.org/10.1101/2023.06.09.23291213], that included repeated samples. Furthermore, we developed new techniques to determine temporal changes from similar quantitative data in young adults.^16^ These two developments gave the opportunity to extend the evidence from young adults into older adults in their 40s and beyond.

Obesity is a causal risk factor for metabolic morbidity^17–21^ and powerful interventions (low-energy diet) and new drugs (e.g. GLP-1 receptor agonists) exist to modify it.^22^^,^^23^ Weight change and disease risk have been studied in the UK Biobank before, but based on semi-quantitative questionnaire data without trajectory analyses.^24^ Whereas cross-sectional and experimental associations between obesity and cardiometabolic diseases are well established,^18^^,^^23^^,^^25^^,^^26^ temporal studies of the co-evolution of obesity and metabolic dysfunction can yield unexpected insight. For example, we identified divergent dynamic metabolic relationships between waist-hip ratio and body mass index using quantitative longitudinal data^27^—we found that widening waist tracks ageing per se better than body mass. The new temporal evidence helps in answering questions on how to define adverse adiposity^28^ and what is the role of body composition regarding disease burden and health care costs in real-world human populations.^21^

Based on our previous study in young adults,^16^ we hypothesized that weight change in older adults is also associated with substantial longitudinal changes in the circulating metabolome. In this study, we characterize the temporal relationships between age, weight change and metabolic health in 10 104 participants of the UK Biobank. The design includes three arms (stable, weight loss and weight gain) that were matched at baseline. We investigated 220 variables that included anthropometric indicators, clinical biomarkers and NMR metabolomics. Our results comprise a useful resource to understand how the metabolome changes due to ageing in human populations during the period where age-associated morbidity starts to rise dramatically.

## Methods

**Figure 1.**
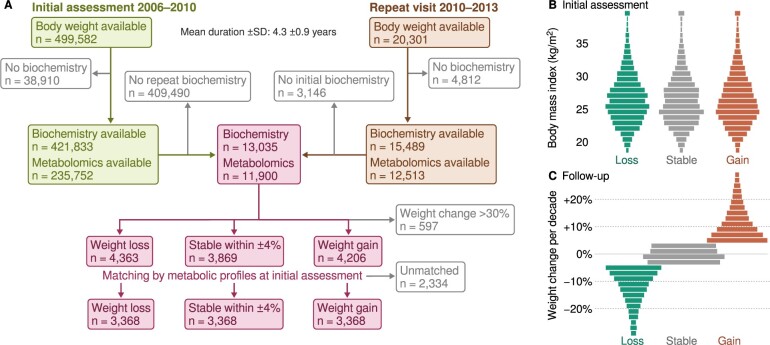
Participant selection and classification. (A) Flow chart of study design. Weight change was reported as percent per decade to equalize the effect from differing duration between assessments. The primary selection criterion was based on the proportional change in body weight: we use a strict definition for a stable body weight to stay within +/– 4% of baseline weight per decade. (B) The secondary selection by multivariate metabolic profiling was performed to control for baseline confounders. We required that there be no substantial difference between the three groups in any of the pre-selected matching variables (visualized in Supplementary Figure S1, available as Supplementary data at *IJE* online) and that the three final groups be of equal size. (C) The majority of the selected participants showed a moderate change (<10% in either direction) or stable weight. In the weight loss subgroup, median weight change was –9.9% per decade. In the weight gain subgroup, median weight increase was +9.2% per decade

The selection of study subjects is described in Figure 1. The data used in this study were obtained from the UK Biobank in September 2023 under the project #72486. We identified 13 035 subjects with sufficient data available and who had made two visits.^29^ We divided the individuals into three weight change categories (loss < –4% ≤ stable ≤ +4% < gain). The 4% cutoff was chosen as the smallest value that provided balanced category sizes. The final subgroups (loss: *n* = 3368, stable: *n* = 3368, gain: *n* = 3368, total: *n* = 10 104) were chosen using nearest neighbour matching across multiple metabolic measures^16^; see also Supplementary Figure S1 (available as Supplementary data at *IJE* online).

The UK Biobank biochemistry panel comprises 30 blood-based and four urine-based measures: see UK Biobank showcase at [https://biobank.ndph.ox.ac.uk/showcase/]. Metabolomics covers 251 blood-based measures, of which eight overlap with the biochemistry panel.^9^^,^^25^^,^^30^ If overlapping, biochemistry assays were chosen due to fewer missing values, except for NMR-based low-density lipoprotein (LDL) and high-density lipoprotein (HDL) cholesterol that were chosen to ensure compatibility with other NMR-based lipoprotein measures. After excluding uninformative variables, 211 biochemical (and nine physiological) measures were included (Supplementary Table S1). Missing values were imputed by nearest neighbours with exact numbers of usable values included in Supplementary Tables S3–S5 (available as Supplementary data at *IJE* online).

Samples that are collected and measured years apart are likely to suffer from technical biases due to updates in handling protocols, different storage periods and the evolution of analytical technologies. For this reason, we derived a calibrated version of the longitudinal dataset for statistical analyses using a multivariate matching algorithm.^16^ The calibration coefficients are listed in Supplementary Table S1 (available as Supplementary data at *IJE* online). The table also includes population-wide median absolute deviations that were used for standardized illustrations of the results.

### Statistical analyses

Longitudinal changes were calculated by subtracting the value at the initial assessment from the value at the repeat visit, for each metabolic measure and participant, respectively. Subgroup comparisons were conducted by calculating the median of the longitudinal change. In practice, the rates of change were rank transformed, then subgroup means of the ranks were calculated, and last the means were reverted back to the original scale and location. This approach produced accurate medians for well-behaving distributions, but it is also robust for variables with duplicated values. *P*-values, standard errors and confidence intervals were estimated using bootstrapping. We chose the cutoff *P *<0.0001 to highlight the most robust findings.^31^

Principal component analysis was employed to assess systemic metabolic differences between the subgroups. The biochemical data were highly collinear, which may bias the results (e.g. numerous collinear lipid variables may lead to overemphasis of lipid metabolism). For this reason, we prioritized a subset of variables with mutual covariance below 50%. First, we ordered the variables according to their associations with age and body mass index. Next, the ordered list was traversed and variables were selected only if they were not strongly correlated with any of the already selected variables (R^2^ <50%). This procedure resulted in a final set of 22 inputs for the model (Supplementary Table S2, available as Supplementary data at *IJE* online).

The first two components were trained with a subset of the UK Biobank with standard biochemistry and metabolomics available (*n* = 227 272 excluding the subgroup members). For the final visualization, the data from subgroup members were projected onto the pre-trained components and the mean coordinates were set as the subgroup profile locations. Two-dimensional Gaussian approximations for 95% confidence envelopes were calculated according to the standard deviations of the horizontal and vertical coordinates.

## Results

The characteristics of participants are listed in Supplementary Tables S3 and S4 (available as Supplementary data at *IJE* online) and subgroup comparisons are illustrated in Figure 1B and Supplementary Figure S1 (available as Supplementary data at *IJE* online). All rates of change have been standardized to mean absolute deviations per decade for compatibility with earlier studies. Highlighted findings in the text satisfy *P *<0.0001 unless specified otherwise. Full results are available in Supplementary Table S5 (available as Supplementary data at *IJE* online).

### Ageing without weight change

**Figure 2.**
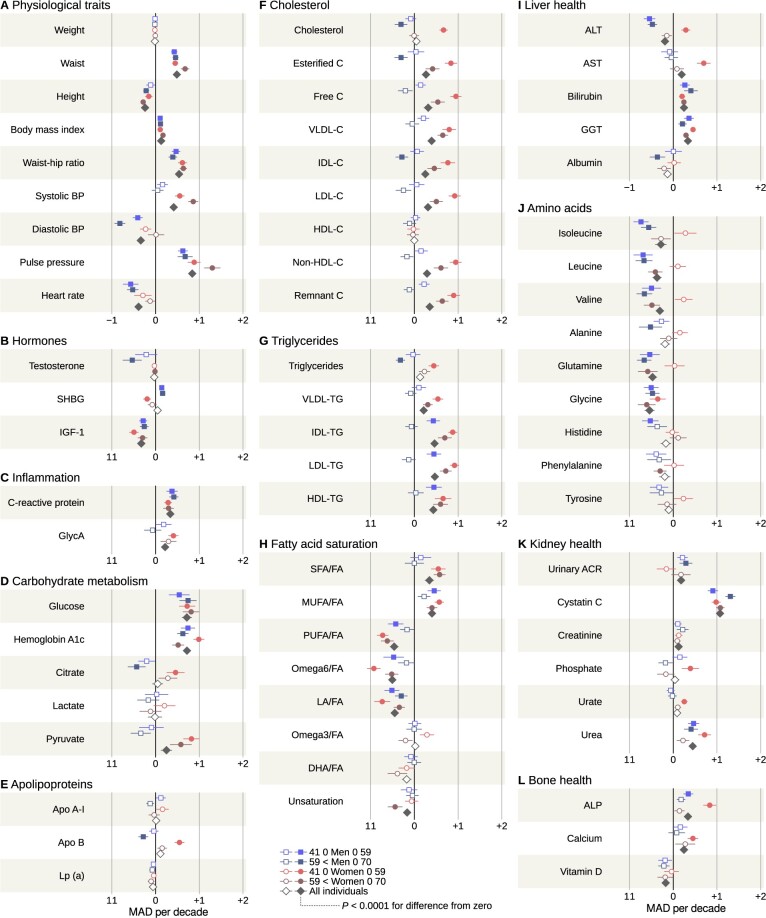
Metabolic changes in the stable weight subgroup stratified by age and sex. Median rates per decade and 95% confidence intervals are illustrated. Filled vs open symbols are indicative of *P* < 0.0001 for divergence from zero change (please see the legend below Plot H). BP, blood pressure; SHBG, sex hormone binding globulin; IGF-1, insulin-like growth factor; GlycA, glycoprotein acetyls; VLDL, very low density lipoprotein; IDL, intermediate density lipoprotein; LDL, low density lipoprotein; HDL, high density lipoprotein; C, cholesterol (Plot F only); FA, fatty acids; SFA, saturated fatty acids; MUFA, monounsaturated fatty acids; PUFA, polyunsaturated fatty acids; LA, linoleic acid; DHA, docosahexaenoic acid; ALT, alanine aminotransferase; AST, aspartate aminotransferase; GGT, gamma-glutamyl transferase; ACR, urinary albumin-creatinine ratio; ALP, alkaline phosphatase; MAD, median absolute deviation of the study population

In the stable subgroup, ageing manifested as widening waist (+4.4 cm per decade) across age and sex strata (Figure 2). There was also an increase in pulse pressure (+7.2 mmHg). Other trends that were consistent across age and sex included decreases in insulin-like growth hormone 1 (–1.19 nmol/L), ratio of linoleic acid (–1.01%age points) and glycine (–19.1 µmol/L), and increases in C-reactive protein (+0.282 mg/L), glucose (+0.254 mmol/L), gamma-glutamyl transferase (+3.22 U/L) and cystatin-C (+94.2 µg/L).

The two male age strata exhibited similar changes for most variables, with notable divergence observed for diastolic blood pressure (–2.8 mmHg per decade for younger vs –5.5 mmHg per decade for older), LDL triglycerides (+10.7 vs –3.1 µmol/L) and cystatin-C (+79.9 vs +115.0 µg/L). Statin use was prevalent in older men (>40%) and a plausible contributor to the age stratification of lipoprotein-related variables (Supplementary Figure S2, available as Supplementary data at *IJE* online).

Women exhibited divergence based on baseline age that was qualitatively different from men. Faster changes in the younger compared with the older stratum were observed for haemoglobin A1c (+2.53 vs +1.32 mmol/mol), apolipoprotein B (+87.2 vs +24.1 mg/L), total cholesterol (+0.506 vs –0.005 mmol/L), aspartate amino transferase (+2.65 vs +0.32 U/L) and alkaline phosphatase (+11.8 vs +2.0 U/L). The older stratum showed a faster increase in systolic blood pressure (+6.8 vs +10.6 mmHg) and a faster decrease in valine (+6.5 vs –13.2 µmol/L). No estradiol was detectable in women over 59 (Supplementary Figure S3, available as Supplementary data at *IJE* online). The prevalence of hormone replacement therapy was below 10% (Supplementary Figure S2, available as Supplementary data at *IJE* online).

### Weight gain versus weight loss

**Figure 3.**
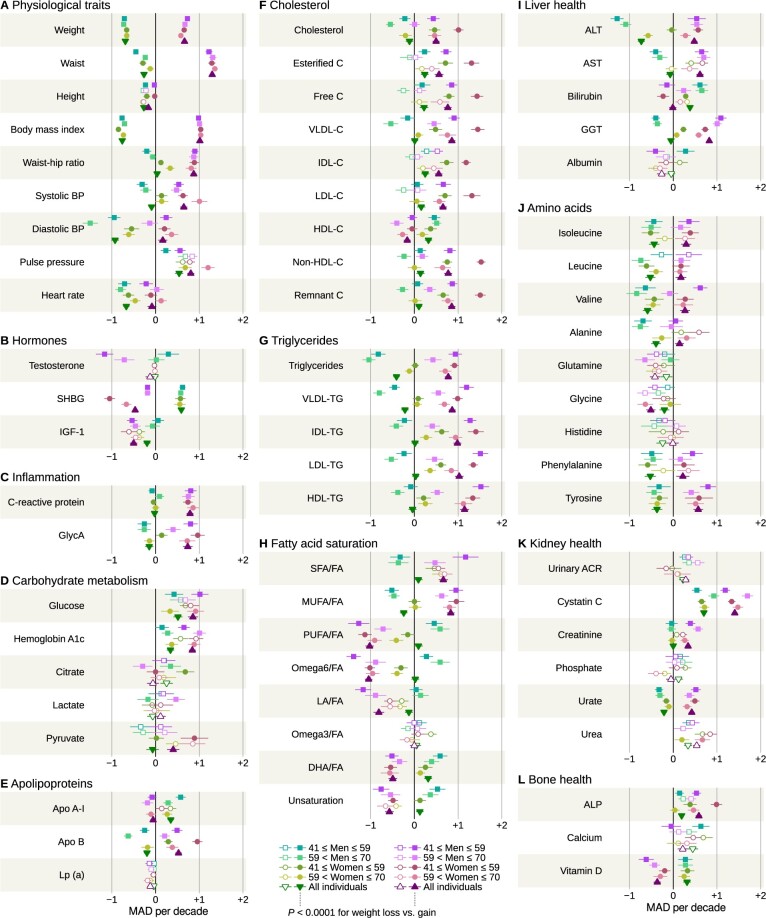
Metabolic changes in the weight loss and gain subgroups stratified by age and sex. Median rates per decade and 95% confidence intervals are illustrated. Filled vs open symbols are indicative of *P* <0.0001 for divergence from zero change (please see the legend below Plot H). BP, blood pressure; SHBG, sex hormone binding globulin; IGF-1, insulin-like growth factor; GlycA, glycoprotein acetyls; VLDL, very low density lipoprotein; IDL, intermediate density lipoprotein; LDL, low density lipoprotein; HDL, high density lipoprotein; C, cholesterol (Plot F only); FA, fatty acids; SFA, saturated fatty acids; MUFA, monounsaturated fatty acids; PUFA, polyunsaturated fatty acids; LA, linoleic acid; DHA, docosahexaenoic acid; ALT, alanine aminotransferase; AST, aspartate aminotransferase; GGT, gamma-glutamyl transferase; ACR, urinary albumin-creatinine ratio; ALP, alkaline phosphatase; MAD, median absolute deviation of the study population

By design, substantial divergence was expected for body weight (–9.9% vs +9.2% per decade) and waist (–2.4 vs +11.7 cm). Notably, waist-hip ratio did not decrease in women who lost weight (Figure 3A). Whereas decreasing blood pressure and heart rate were strongly associated with decreasing body weight, there was less divergence in pulse pressure (all subgroups and age and sex strata showed an increase).

Weight gain was associated with adverse changes for most biochemical measures. Faster increase was seen for C-reactive protein (–0.020 mg/L per decade with weight loss vs +0.658 mg/L per decade with weight gain), haemoglobin A1c (+0.87 vs +2.14 mmol/mol), LDL cholesterol (+0.045 vs +0.190 mmol/L), gamma-glutamyl transferase (–0.51 vs +7.86 U/L), cystatin C (+62 vs +124 µg/L) and alkaline phosphatase (+2.71 vs +8.26 U/L). Consistent directional divergence along body weight were seen for triglycerides (–0.21 vs +0.39 mmol/L) and multiple amino acids such as tyrosine (–3.31 vs +5.10 µmol/L). Urate was also associated with weight change (–11.6 vs +23.1 µmol/L). Inverse weight gain associations were observed for sex hormone binding globulin (+8.7 vs –7.0 nmol/L), polyunsaturated fatty acid ratio (+0.22 vs –2.42%age points) and vitamin D (+4.7 vs –5.5 nmol/L).

Stronger weight associations with citrate and lactate were observed in men over 59 but not in those under 59. Conversely, serum albumin was associated with weight change only in the younger stratum. Sex hormone binding globulin plummeted in women under 59 compared with older women and men, and alkaline phosphatase increased the fastest in women under 59 who gained weight.

We summarized the similarity of temporal patterns between the sexes by calculating the correlation between the observed slopes from univariate analyses (Supplementary Figure S4, available as Supplementary data at *IJE* online). Weight gain was associated with a similar response in men and women for both the younger (R^2^ = 74.1%) and older strata (R^2^ = 72.1%). However, the stable and weight loss subgroups exhibited more sex differences (lower correlation) with correlations between 26.3% and 43.1%.

### Weight change and metabolic profiles

**Figure 4.**
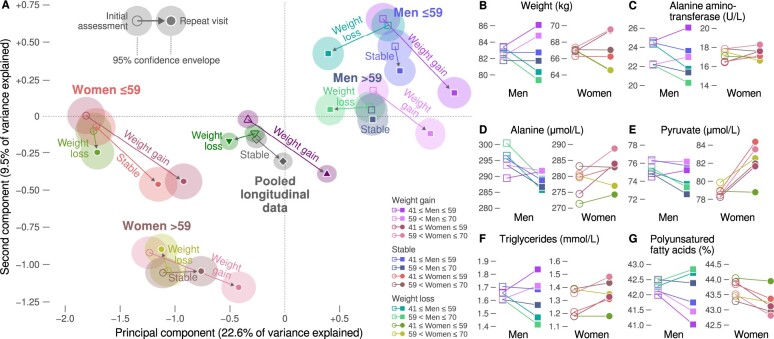
Summary of metabolic changes by subgroup stratified by age and sex. Open and filled symbols indicate the baseline and follow-up visits, respectively. (A) The principal components were calculated from those UK Biobank participants with full metabolic data available (training set, *n* = 227 272) but who were not included in the longitudinal dataset. The plot was created by projecting the longitudinal dataset (*n* = 10 104) onto the principal components. Symbol locations were determined according to the mean first and second component scores. (B–G) Univariate visualizations of longitudinal changes. The first column represents the value at the initial assessment and the second column is the value at the repeat visit

To identify deeper systemic patterns of metabolic change, we trained a principal component decomposition with the full biochemical UK Biobank dataset (*n* = 227 272) and then projected the weight subgroup members onto the decomposition according to their metabolic profiles (additional details in Methods). The projections are shown in Figure 4A. Separate projections were made for the initial and repeat visits, and these are connected pairwise by arrows. There is a large gap between men and women which reflects the fundamental baseline sex differences in most metabolic measures. Weight change is the second evident phenomenon, particularly in men. The transitions create a visual pattern that resembles a Christmas tree, with ‘pure’ ageing visible as the drift downwards of the stable category and weight change manifesting as branches to the left (weight loss) and right (weight gain) which are replicated across the two age strata.

By design, the weight subgroups were indistinguishable at baseline (confidence envelopes overlap in Figure 4A, see also Figure 4B). However, the female age strata had substantially different baseline profiles, as indicated by the distance on the canvas between the older and younger women (male profiles are clustered closer to each other). Furthermore, weight loss in men was associated with a transition towards lower left, but this did not occur in women. Both the stable and weight gain female subgroups progressed in the lower right direction, and the weight loss subgroup stayed close to the starting position for both age strata. We consider the lower right transition as metabolically adverse, since this is the direction of weight gain arrows for each sex and age subgroup.

Selected examples of univariate trajectories underlying the principal component analysis are depicted in Figure 4. Alanine aminotransferase and its reaction target alanine and catalyzed product pyruvate were among the most qualitatively different measures between the sexes (Figure 4C–E). Cholesterol-related measures, including LDL cholesterol, increased faster in women compared with men (Supplementary Figure S4, available as Supplementary data at *IJE* online). Further, triglyceride measures and fatty acid ratios in men were divergent between weight subgroups but less so in women. Both stable and weight gain subgroups in women were associated with comparable increases in triglycerides (Figure 4F) and decreases in polyunsaturated fatty acid ratio (Figure 4F). Female weight loss was associated with flat trends in these measures. Regarding men, the slopes of these measures were similar to women only in the weight gain subgroup. In particular, male weight loss was associated with substantial decreases in triglycerides and increases in polyunsaturated fatty acid ratio, unlike the flat trends in women.

## Discussion

We report age-associated longitudinal changes in over 200 metabolic measures in their original concentration units for 10 104 UK Biobank participants between the ages of 41 and 70. Such statistics have not been previously reported for most of the measures (not possible with cross-sectional data). We observed substantial divergence associated with weight loss, weight gain, sex and baseline age, and temporal associations were dissected for multiple molecular measures such as metabolically important hormones and protein biomarkers, pyruvate and other indicators of carbohydrate metabolism, detailed lipoprotein measures, polyunsaturated fatty acids, amino acids, tissue-specific biomarkers and vitamin D.

The new results for older individuals fit well with earlier findings in young adults.^16^ In both studies, waist-hip ratio increased even without weight gain, which likely reflects changes in body composition.^32^ Ageing was associated with increasing inflammation, declining glucose homeostasis and increasing blood pressure, which contribute towards higher cardiovascular and diabetes risk.^4^^,^^33^ These changes were reduced with weight loss. Weight gain was associated with shifts towards hyperglycaemia, high triglycerides with an imbalance between very low density lipoproteins (VLDL) and HDL, worsening liver health, increased branched-chain amino acids and other hallmarks of the metabolic syndrome.^34^ The combined results from younger and older individuals indicate that metabolic benefits associated with weight reduction are reproducible across a wide age range.

Women aged differently compared with men. Menopause is the plausible explanation, since it affects only women and is characterized by hormonal and metabolic changes.^6^^,^^35^^,^^36^ The cross-sectional study by Auro *et al*.^6^ found robust signals for esterified cholesterol, multiple lipoprotein measures and polyunsaturated fatty acids, which were higher in post- compared with pre-menopausal women (body weight or composition was not investigated). Our results show that this transition is faster during the typical menopause age range and that these measures are robustly associated with weight change (or confounded, depending on perspective). Wang *et al*. reported longitudinal menopause-associated metabolic changes in a cohort of UK women.^36^ The main observations of adverse atherogenic changes regarding lipoprotein cholesterol and triglycerides were similar to the findings from the UK Biobank.

Some of the new results did not match with previous findings. Increased glutamine and glycine were reported in the other cohorts,^6^^,^^16^^,^^36^ whereas negative trends were observed in the UK Biobank. Glutamine was calibrated unusually low for the repeat visit (scale factor of 0.94, see Supplementary Table S1, available as Supplementary data at *IJE* online), which may explain the difference. Glycine was not affected by batch effects (scale factor of 0.994) and was consistently decreasing across age and sex strata, which excludes obvious technical explanations. Decreased glycine in late life makes sense in light of anti-ageing experiments^37^ and human observations.^38^ Serum albumin increase was associated previously with menopause,^36^ but we did not see an increase in the younger female stratum. We did observe a decrease in older men, which is compatible with literature.^39^

The sex difference in the alanine-pyruvate axis is a curiosity (Figure 4C–E). Circulating concentrations may not reflect cellular processes in tissues and this evidence must be interpreted with caution. Nevertheless, the disconnect between weight change and alanine aminotransferase in women could mean that the Cahill cycle may be one a key pathway disrupted by menopause, or it could be one of the key regulatory differences between male and female metabolism.^34^^,^^40^

The connection between body mass and mortality is U-shaped^19^ and is complicated by body composition.^32^ This study has a unique perspective, since we dissected the precise associations with weight change. Although we cannot establish causality based on the data at hand, it is safe to assume that body weight has a considerable impact on metabolic health, based on the literature.^18^^,^^19^^,^^41^ Our conclusion is that maintaining stable weight may not be enough for the female population over 40 to avoid the rapid decline in metabolic health, and even with weight loss, improvement is unlikely. Men appear to respond consistently to weight change even when older, which highlights the need and opportunities to address modifiable cardiometabolic risk factors such as obesity from a sex-specific viewpoint.

Our study is statistically robust due to the large size of the UK Biobank. Nevertheless, the UK Biobank is a volunteer cohort and generalization to the entire population or other countries should be conservative. The longitudinally matched design allowed us to simplify statistical analyses and to reduce confounding from sample selection between the subgroups. The divergence between male age strata (and difference to women) may have been affected by statin use, and results regarding LDL cholesterol should be interpreted with caution.^42^ Female hormone replacement therapy increases triglycerides and decreases LDL cholesterol and multiple amino acids,^43^ but it is unlikely to skew the averages due to the low prevalence of less than 10%. Previous analysis of the UK Biobank suggests that medications have a modest effect on the full metabolic profile.^25^

This study is limited to the description of temporal associations from a population perspective and the results should not be seen as causal evidence. Weight gain or loss can be caused by severe diseases such as cancer, injuries or other life events. We did not detect common drivers for weight change in the diagnostic records (data not shown). The weight remained within ±10% per decade (or ±1% per year) for two-thirds of the subjects, which is a plausible rate for a modern population, and therefore we are confident that the results presented here reflect the gradual ageing phenomenon rather than specific extreme events.

## Ethics approval

This study did not involve recruitment of study subjects or new biomedical experiments. The ethics statement for the UK Biobank is available online [https://www.ukbiobank.ac.uk/learn-more-about-uk-biobank/about-us/ethics].

## Supplementary Material

dyae055_Supplementary_Data

## Data Availability

The UK Biobank data are available to the public [https://www.ukbiobank.ac.uk/].
