# Supplementary material for: Influence of age and sex on longitudinal metabolic profiles and body weight trajectories in the UK Biobank
Source: Int J Epidemiol. 2024 Apr 19;53(3):dyae055. doi: 10.1093/ije/dyae055 (PMC11031410; doi:10.1093/ije/dyae055)
Supplement: dyae055_Supplementary_Data [file dyae055_supplementary_data.zip › ije-2023-12-1531-File006.pdf]

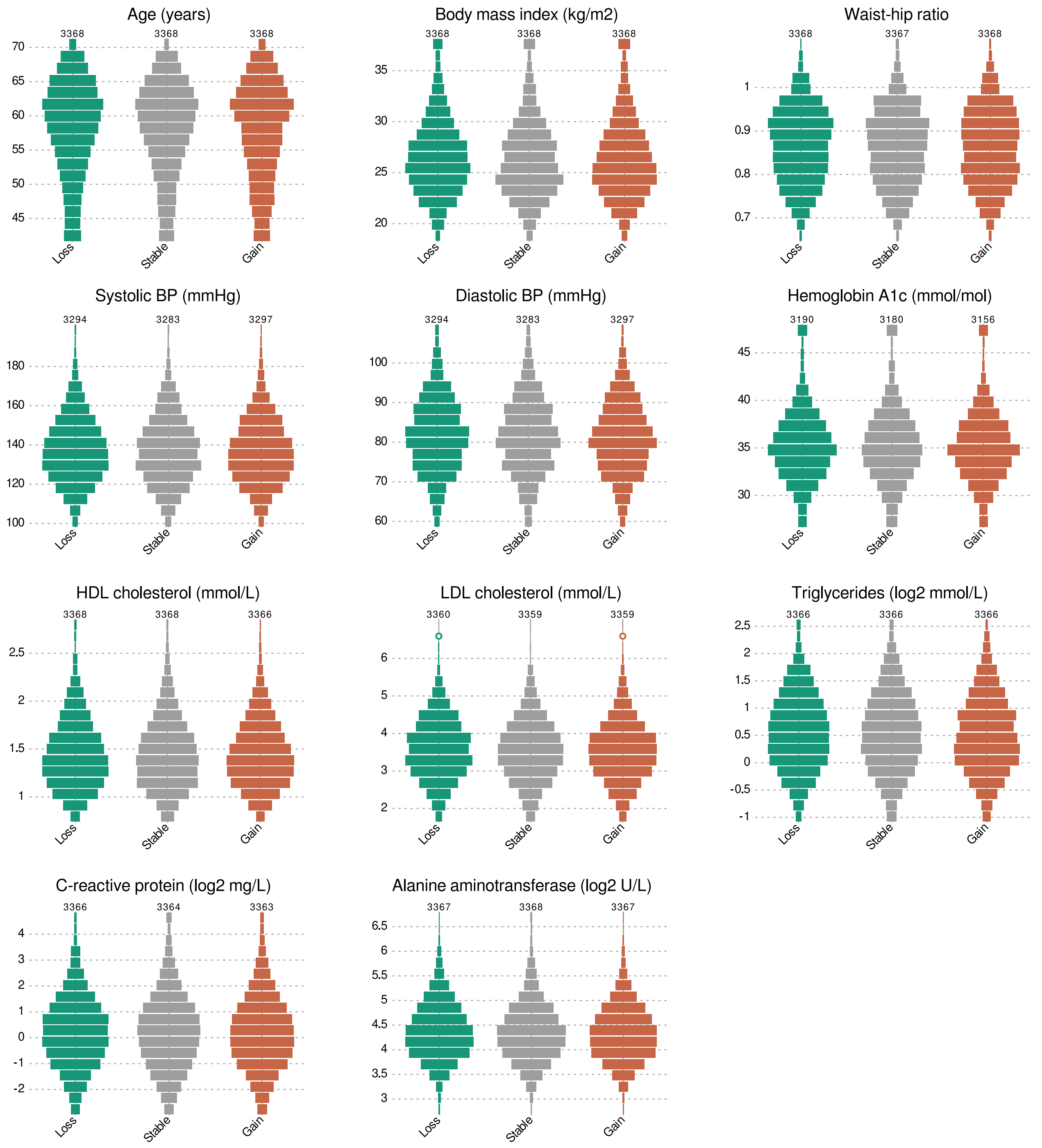

Figure S1: Metabolic measures that were used for nearest neighbor matching between weight subgroups. The plots show the value histograms for the final matched subsets. None of the variables showed substantial differences between subgroups ($P > 0.0001$).

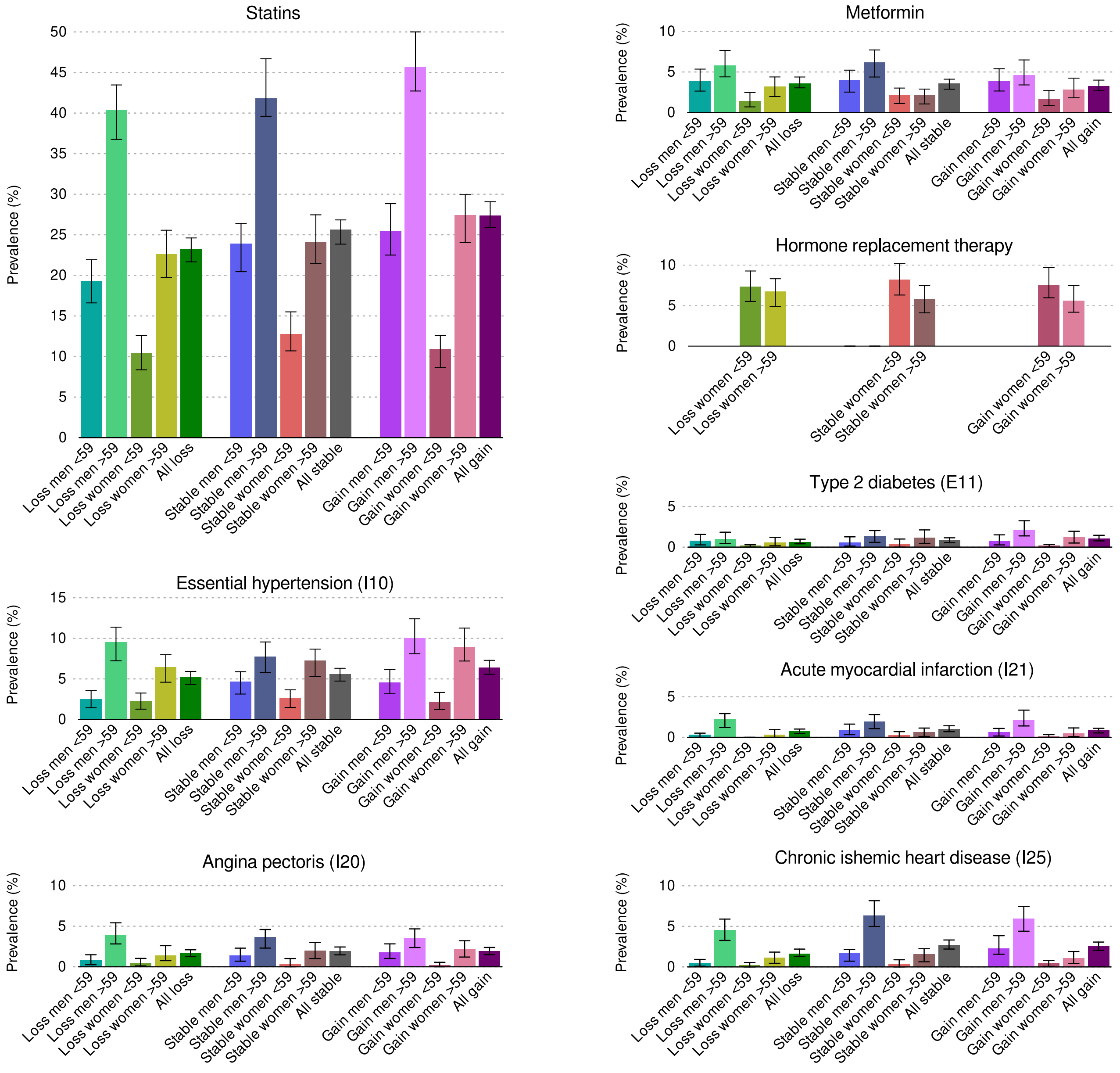

Figure S2: Comparison of typical cardiometabolic medications and diagnostic codes (ICD-10) between weight subgroups. Apart from statins (and the high low-density lipoprotein cholesterol that they target), most potential confounders had low prevalence in the study participants. The coverage of the UK Biobank diagnostic data is not perfect, therefore the prevalences may be under-estimated. Nevertheless, the three main subgroups (weight loss, stable and weight gain) did not differ substantially despite the heavy age and sex stratification, which means that the metabolic matching at baseline was successful. By extension, we conclude that selection bias by UK Biobank is likely to affect each subgroup equally and can be dismissed when comparing weight change within the same age and sex strata.

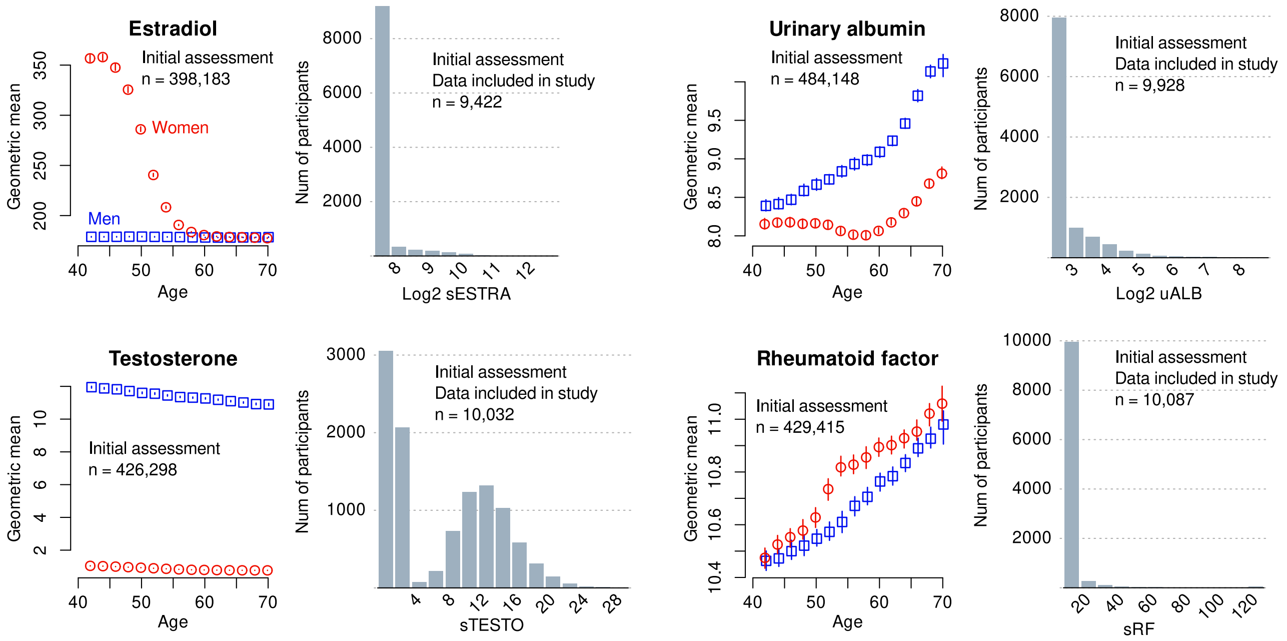

Figure S3: Examples of variables that had atypical distributions. Estradiol, urinary albumin and rheumatoid factor were excluded from the main text (but included in the results) due to the low accuracy of the assays. Most participants had concentrations below the detection limit for these measures and thus effectively the same low value from a practical perspective. Testosterone assay was accurate enough for men, however, due to the dramatic sex difference, it was excluded from multi-variate analyses as this type of bimodal distribution would have an oversized effect on statistical results.

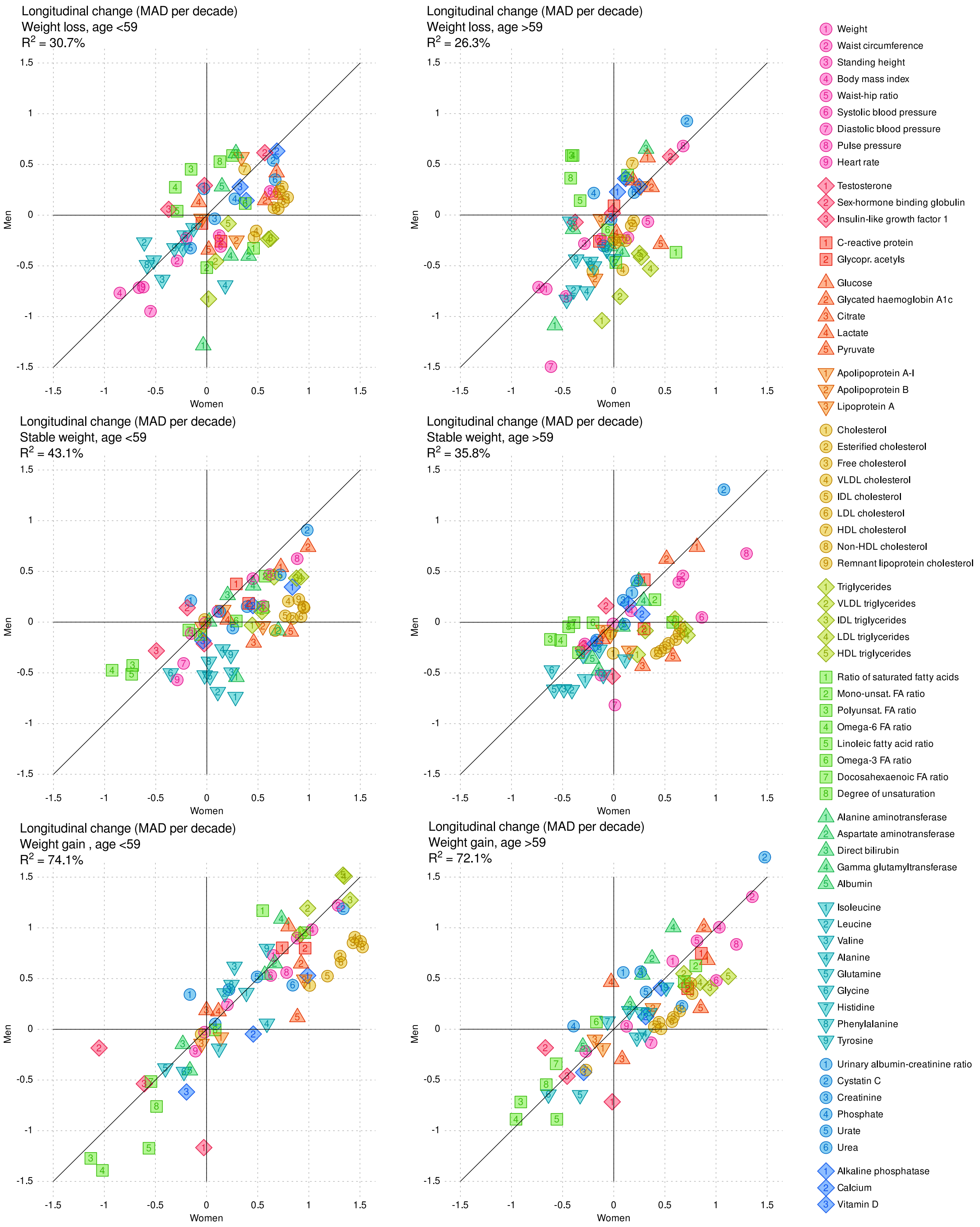

Figure S4: Comparison of longitudinal slopes between men and women (alternative visualization to Figures 2 and 3 in the main text). Abbreviations: VLDL, very low density lipoprotein; IDL, intermediate density lipoprotein; LDL, low density lipoprotein; HDL, high density lipoprotein; FA, fatty acids; MAD, mean absolute deviation.
